# Maternal *Toxoplasma gondii* infection affects proliferation, differentiation and cell cycle regulation of retinal neural progenitor cells in mouse embryo

**DOI:** 10.3389/fncel.2023.1211446

**Published:** 2023-07-21

**Authors:** Viviane Souza de Campos, Camila Feitosa Magalhães, Barbara Gomes da Rosa, Carolina Moreira dos Santos, Lucianne Fragel-Madeira, Danniel Pereira Figueiredo, Karin C. Calaza, Daniel Adesse

**Affiliations:** ^1^Laboratório de Neurobiologia da Retina, Instituto de Biologia, Universidade Federal Fluminense, Niterói, Brazil; ^2^Laboratório de Biologia Estrutural, Instituto Oswaldo Cruz, Fiocruz, Rio de Janeiro, Brazil; ^3^Laboratório de Desenvolvimento e Regeneração Neural, Instituto de Biologia, Universidade Federal Fluminense, Niterói, Brazil; ^4^Department of Biochemistry and Molecular Biology, Miller School of Medicine, University of Miami, Miami, FL, United States

**Keywords:** *Toxoplasma gondii*, congenital toxoplasmosis, retinal neurogenesis, retinal progenitor cells, cell cycle proteins, TORCH infections, cyclins

## Abstract

**Background:**

Toxoplasmosis affects one third of the world population and has the protozoan *Toxoplasma gondii* as etiological agent. Congenital toxoplasmosis (CT) can cause severe damage to the fetus, including miscarriages, intracranial calcification, hydrocephalus and retinochoroiditis. Severity of CT depends on the gestational period in which infection occurs, and alterations at the cellular level during retinal development have been reported. In this study, we proposed a mouse CT model to investigate the impact of infection on retinal development.

**Methods:**

Pregnant females of pigmented C57BL/6 strain mice were infected intragastrically with two *T. gondii* cysts (ME49 strain) at embryonic day 10 (E10), and the offspring were analyzed at E18.

**Results:**

Infected embryos had significantly smaller body sizes and weights than the PBS-treated controls, indicating that embryonic development was affected. In the retina, a significant increase in the number of Ki-67-positive cells (marker of proliferating cells) was found in the apical region of the NBL of infected mice compared to the control. Supporting this, cell cycle proteins Cyclin D3, Cdk6 and pChK2 were significantly altered in infected retinas. Interestingly, the immunohistochemical analysis showed a significant increase in the population of β-III-tubulin-positive cells, one of the earliest markers of neuronal differentiation.

**Conclusions:**

Our data suggests that CT affects cell cycle progression in retinal progenitor cells, possibly inducing the arrest of these cells at G2/M phase. Such alterations could influence the differentiation, anticipating/increasing neuronal maturation, and therefore leading to abnormal retinal formation. Our model mimics important events observed in ocular CT.

## Introduction

Vision is the sense that enables us to see everything around us, including at a distance, becoming an adaptively useful sense for animals (Lent, 2002). The vertebrate retina is a tissue organized in nuclear layers interspersed by plexuses, consisting of six main cell types, including five neuronal classes and one main glial cell type (Lent, 2002). Retinal development involves proliferation, determination of cell fate, migration, differentiation, apoptosis and synaptogenesis (Wong, 2006). These events, and the period in which they occur, determine the generation of different cell types, their proportion and distribution in the layers, and finally their connections (Wong, 2006).

During vertebrate retinal development, cell proliferation and cell cycle exit are highly coordinated events essential for retinal formation (Martins and Pearson, 2008). In the proliferative stage, cell cycle is mainly controlled by different cyclins, which activate specifically cyclin-dependent kinases. Cycle progression relies on variations in the concentration of each specific cyclin, leading to a cascade of molecular changes in the cell, which, if altered can be developmentally harmful (Bilitou and Ohnuma, 2010). Mouse retinal neurogenesis, the cell birth/exit of cell cycle, can be separated into two periods. The first wave occurs mainly during the embryonic phase, with ganglion, horizontal, cone and amacrine cell genesis, from E11 to E18. The second period occurs in the postnatal phase, peaking between the day of birth and post-natal day seven (P7), with rods, bipolars and Müller’s glia exiting the cell cycle. The correct development of the retina ensures the functioning of its mature circuitry. The impact of vision deficit/blindness in people’s lives intensify the importance of understanding environmental factors capable of interfering in and damaging retinal development. Several intrinsic and extrinsic factors act on the developmental events mentioned above, and changes caused by insults during pregnancy can be devastating to the retina.

Toxoplasmosis, which affects one third of the world population and has the protozoan *Toxoplasma gondii* as its etiological agent, is a zoonosis of great interest in the field of public health. *T. gondii* is an opportunistic obligate intracellular protozoan parasite, capable of infecting all nucleated cells of warm-blooded animals, thus reaching different tissues of the organism. Moreover, latent cysts are found mostly in muscular and neural niches. Among the infected population, two groups with greater medical importance stand out: immunocompromised individuals and those congenitally infected, in which the most severe manifestations of the disease occur. It is currently known that congenital infection is the most severe form of toxoplasmosis and can occur in the offspring of women who had primary *T. gondii* infection during pregnancy. *In utero* infection can lead to congenital toxoplasmosis (CT), which can cause severe damage to the fetus, such as miscarriage, intracranial calcification, hydrocephalus and retinochoroiditis, depending on the gestational period in which it occurs.

Congenital toxoplasmosis can manifest in the first month of life and can lead to long-term ocular and neurological sequelae in children and adults. Ophthalmologic manifestations are the main sequelae of CT, with retinochoroiditis being the main and most common one, with an estimated incidence of 9 to 31%. Other possible ocular manifestations that can cause visual damage are: strabismus, microphthalmia, cataract, retinal detachment, optic atrophy, iridocyclitis, nystagmus, and glaucoma. Some of these features are apparently correlated with a process of retinochoroiditis, which is then a marker of CT severity (Bollani et al., 1967; Peyron et al., 2019; Yang et al., 2021; Zhou et al., 2023).

In an attempt to mimic some aspects of human CT ocular disease, including parasite invasiveness and pathogenesis, development of some animal models was attempted. The first experimental models to study ocular toxoplasmosis (OT) aimed to describe the pathogenesis of the disease. To analyze the migration of the parasite to the retinal tissue, initially rabbits and hamsters and distinct routes of infection (intracarotid and intraperitoneal) were used (Frenkel, 1955). However, such approaches failed to reproduce the natural routes of host infection. Over the years, there has been an expansion of studies aiming to investigate models of *T. gondii*-induced retinochoroiditis, including non-human primates, cats, rabbits, guinea pigs and mice (Dukaczewska et al., 2015). Changes in the pattern of the retinal cell structure, such as displacement of the retinal pigmented epithelium from the photoreceptor layer and irregular packaging of the retinal layers, have been described in the literature. These changes were noticed in congenitally *T. gondii*-infected mice, where more extensive eye damage was noted as compared to acquired OT (Ashour et al., 2018). Moreover, congenital *T. gondii* infection in mice caused structural damage to the retina of infected offspring, including reduced number of cells in the Outer Nuclear Layer (ONL), mild alteration of Müller glial markers and disorganization of nuclear layers (Lahmar et al., 2010, 2014; Ashour et al., 2018). Despite the severity of congenital ocular toxoplasmosis and the efforts to establish experimental models to recapitulate the human disease in the laboratory settings, very few studies have successfully detected the parasite (either tachyzoites or cysts) in the retinal tissue per se (Lee et al., 1983; Hay et al., 1984; Lahmar et al., 2010; Dukaczewska et al., 2015).

A more recent study comparing mouse strains, parasite strain, stage and inoculum dose, described that adult C57BL/6 mice were more susceptible to oral cyst infection and developed OT rapidly (14 to 21 days post-infection), thus becoming the model that most closely resembles the natural infection (Dukaczewska et al., 2015). In addition, C57BL/6 is also a more adequate model for the study of retinal development since it is not an albino animal. The pigmented epithelium is necessary for the normal development and maintenance of the mouse neural retina, with albino showing several neuroretina defects (Raymond and Jackson, 1995). Thus, C57BL/6 represents a better model to study the specific effect of *T. gondii* infection in retinal development, and a rodent model that more closely mirrors the visual system of most of the human population.

Despite these previous observations, no studies have addressed whether the damages found in congenital OT arise from changes in the biology of the retinal progenitor cell populations, including their proliferation and differentiation rates during development, thus demonstrating the need to establish proper models to allow such investigations. In the present work, we studied the impact of vertical *T. gondii* transmission in a relevant mouse model of retinal development, focusing particularly on the proliferation and differentiation of retinal progenitor cells. We observed that infection of C57BL/6 pregnant females at E10 generated gross abnormalities, including smaller embryos. Retinal neurogenesis was impaired, with changes in retinal proliferation and differentiation as well as in the expression of cell cycle proteins.

## Materials and methods

### *T. gondii* maintenance and isolation

ME49 strain parasites were maintained in C57BL/6 mice. For tissue cysts isolation, whole brains of previously infected animals were collected and macerated with needles of different gauges, starting at 18G, 21G, 22G, 23G, and 26 G, in the presence of PBS. The determination of cyst number was performed using a phase contrast light microscope: brain macerates were resuspended in 2 ml of PBS and 50 μL of the cyst suspension was placed on a glass microscope slide and covered with a 22 × 22 mm glass coverslip and the number of cysts was counted visually in the whole area of the coverslip, as described previously (Hermes et al., 2008; Estato et al., 2018). The observed number of cysts in the whole slide was multiplied by 40, to obtain the number of cysts per milliliter and at least 3 aliquots of brain homogenates were evaluated. Each cyst-containing aliquot in PBS was first examined under inverted phase contrast microscope in a 96-well plate well, to ensure the exact number of structures being handled.

### Mouse model of congenital toxoplasmosis

To assess the impact of congenital infection on mouse retinal development we have used C57BL/6 mice, supplied by the Instituto de Ciência e Tecnologia em Biomodelos (ICTB/Fiocruz). The use of the C57BL/6 strain mice was approved by the Ethics Committee for the Use of Laboratory Animals of the Instituto Oswaldo Cruz, license number L-048/2015. Mating was performed by leaving two female mice in a cage in which bedding was previously used for a male mouse for 72 h, in an adaptation of the protocol by Stiles et al. (2013) to synchronize the estrus cycle. Two females were added to each cage with one male stayed for 24 hours. Then, females were removed and pregnancy started to be counted (embryonic day 1, E1). Females were weighted at E0.5 and E10 and those that gained more than 10% weight were assumed to be pregnant. Those animals were then marked and inoculated intragastrically (via oral gavage) with PBS or *T. gondii* with 2 cysts in total volume of 200 μL. Then, the females were placed in individual cages and kept with environmental enrichment program (red acrylic igloos, sterile toilet paper for nesting, etc.) until the endpoints. Animals from both groups were euthanized at E18 or E20 via lethal intraperitoneal injection with pentobarbital. The embryos were removed from the uterine cavity, weighted, photographed, and euthanized by decapitation using surgical scissors and then the eyeballs were removed for further analysis. Otherwise, we followed pregnancies until the day of birth (P0). For this study, a total of 56 pregnant females were used as summarized in Table 1 and in detail in Supplementary Table 1.

**TABLE 1:** Number of pregnant female C57BL/6 mice used in this study.

Inoculum	E18	E20	P0
PBS	9	1	13
2 cysts	9	2	13
5 cysts	–	–	7
10 cysts	–	–	2

### Tissue processing

Eyeballs of control and infected offspring were fixed in 4% paraformaldehyde for 30 min, washed in phosphate buffer three times and subjected to a sucrose gradient (15 and 30%) for cryoprotection. The samples were oriented under a dissection microscope in an aluminum mold, filled with Optimum Cutting Temperature embedding medium (OCT) and frozen using liquid N_2_. Cross-sections (10 μm) were made in a cryostat and collected in Poly-L-lysine-coated glass slides for immunohistochemistry.

### Immunohistochemistry

Sections were washed with PBS and blocked with 5% bovine serum albumin (BSA) in PBS for 1 h, followed by overnight incubation with the primary antibody at 4°C (Supplementary Table 2). Sections were washed three times with PBS and incubated with secondary antibody conjugated to AlexaFluor 488 and AlexaFluor 594 (Thermo Fisher Scientific) for 2 h in the dark. Antibodies were diluted in 0.25% Triton X-100 in PBS. Following washes with PBS, cell nuclei were labeled with DAPI (4’, 6-Diamidino-2-phenylindole; Sigma-Aldrich) at 0.2 μg/mL in PBS for 10 min, washed with PBS and mounted with DABCO (1,4-Diazabicyclo[2.2.2]octane solution) mounting medium with 50% glycerol in PBS (Sigma Aldrich).

### Quantification of immunostaining and cells in retinal sections

Confocal images of retinal slices were acquired using a Plan-Apochromat 63x/1.4 objective with constant intensity settings on the Zeiss 710 Meta Spinning disk laser confocal microscope (Platform for Optical Light Microscopy Gustavo de Oliveira Castro, PLAMOL, UFRJ) or on the ZEISS ELYRA PS.1 Light microscope super-resolution by structured illumination (SIM–Structured Illumination Microscopy) from the National Center for Structural Biology and Bioimaging (CENABIO). Quantifications were performed using ImageJ (NIH) software. Nine microscopic fields from each retina were quantified, separated by central and peripheral region, from six animals, obtained from two litters from control or infected dams. Brn3a and TUJ1-positive cells were counted and normalized by the number of nuclei stained by DAPI. The spatial distribution of TUJ1 labeling was also evaluated. After the images were rotated to the horizontal position, a grid set at 100 μm (suitable according to the average size of the nuclei) was superposed to the image. The number of TUJ1-labeled tiers were counted. For Ki-67 quantification, overall proliferating cells were considered as those in the neuroblastic layer and M-phase mitotic cells as Ki-67-positive cells in the apical part of the neuroblastic layer, normalized by the number of nuclei stained with DAPI in that layer.

### Western blotting

Retinas from E18 animals were dissected from the pigmented epithelium and sclera in CMF (calcium and magnesium free saline; 1.31 M NaCl, 40.9 mM KCl, 9.2 mM Na_2_HPO_4_.7H_2_O, 4.5 mM KH_2_PO_4_, 12.2 mM glucose and ice-cold 9.4 mM NaHCO_3_). Two retinas per animal were dissected and placed in 30 μL of RIPA buffer (150 mM NaCl; 50 mM Tris-base; 5 mM EGTA, 1% Triton; 0.5% sodium deoxycholate; 0.1% SDS) with D- dithiothreitol (Sigma Aldrich, St Louis, MO, USA). Samples were homogenized in a potter, transferred to a microtube (Eppendorf), and centrifuged at 15,000 rpm for 10 min at 4°C. The supernatant was collected and stored at −20°C. Protein concentration was determined by colorimetric assay with bicinchoninic acid (BCA) (Thermo Scientific, Rockford, IL, USA). Thirty or 60 μg of protein were loaded onto 8 or 10% polyacrylamide gels in denaturing sample buffer (0.5 M Tris-HCL/0.4% SDS pH = 6.8; 30% glycerol; 10% SDS; DTT 0.6 M; bromophenol blue 0.06 M) and resolved by electrophoresis. Proteins were transferred to a PVDF membrane through a “Semi-DryTransfer Unit” (Bio-Rad) and membranes were washed with TBS-T (20 mM Tris, 200 mM NaCl and 0.1% Tween 20) and blocked with 5% skim milk diluted in TBS-T for 1 or 2 h. Primary antibodies (Supplementary Table 2) were diluted in 5% milk or BSA and incubated overnight at 4°C. Membranes were then washed in TBS-T and incubated with HRP-labeled secondary antibodies (1:2,000), diluted in 5% milk in TBS-T for 1 h. Anti-α-tubulin was used as loading control. Membranes were exposed to ECL chemiluminescent reagent (#cat. RPN2232; GE Healthcare Life Sciences) and the images were obtained using ChemiDoc (Bio-Rad Laboratories, Hercules, CA, USA) and optical density was analyzed with ImageJ software (version 1.38, NIH, USA).

### Statistical analyses

Statistical analyses were performed using GraphPad Prism 10.0.0 software (GraphPad Software, Inc., San Diego, CA, USA). Data are presented as mean ± standard error of the mean (SEM). Morphometric data of body weight and length were analyzed by Two-way analysis of variance (ANOVA), and differences between groups were evaluated with the Bonferroni post-test. Morphometrical data (embryo body weight and length) was obtained from nine control litters in E18, one control litter for E20, nine infected litters for E18 and 2 infected litters for E20. Placenta weight and diameter were determined from three control and one infected litters at E18. For immunohistochemistry data, twelve E18 embryos from two independent dams per group (PBS control and 2 *T. gondii* cysts) were analyzed. Both eyes were cryosectioned, immunostained, and quantified as described above. Data plotted from each embryo corresponds to the average of their two eyes. For western blotting analyses, both retinas, from three independent E18 fetuses (from three different dams) per experimental group (PBS control and 2 *T. gondii* cysts), were pooled and resolved by SDS-PAGE, in a total of 3 control and 3 infected samples. Data were analyzed with unpaired Student’s *t*-test. The significance level was set at *p* < 0.05.

## Results

### Characterization of a murine model of CT

**FIGURE 1:**
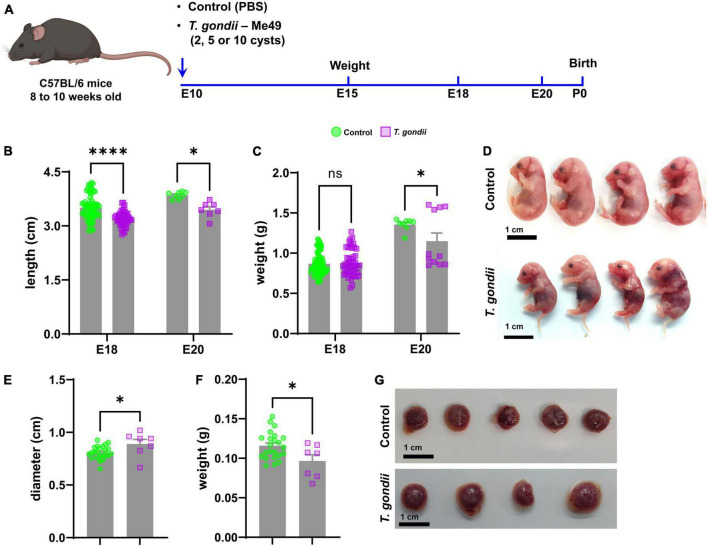
Establishment of a mouse model of congenital toxoplasmosis to assess changes in fetal development. Schematic summary of the experimental model of congenital infection **(A)**. Morphometric analysis at E18 and E20, quantifying embryos’ length **(B)** and weight **(C)** of control (green bar) and infected (lilac bar) embryos. In panel **(D)**, representative photographs obtained from one control and one infected litter at E18. **(E,F)** Show placental diameter and weight at E18, respectively. In panel **(G)**, representative images of placentas from control and *T. gondii*-exposed embryos. Scale bars: 1 cm. *: *p* < 0.05; ****: *p* < 0.00001, ns: not statistically significant, two-Way ANOVA with Bonferroni post-test for panels **(B,C)**; Unpaired Student’s *T*-test for panels **(E,F)**. Each dot on graphs correspond to one embryo from at least 7 independent dams per group.

Initially, we aimed to establish a mouse model that would resemble human vertical transmission of *T. gondii*. We chose C57BL/6 over other albino (e.g., Swiss Webster or Balb/c) mouse strains since their retina possess a pigmented epithelium, which plays a key role in retinal development and physiology (Bilitou and Ohnuma, 2010; Simó et al., 2010). We determined the ideal inoculum to generate ocular CT in C57BL/6 mice. Previous data from our laboratory with SW mice have shown that infection with 25 or 50 ME49 cysts were highly disruptive for embryonic development, with high maternal mortality rates (Hermes et al., 2008). Other studies, focusing on establishing parameters for mouse CT models, showed that infection of C57BL/6 pregnant female mice with 5 or 10 cysts also led to high mortality (Raymond and Jackson, 1995; Lahmar et al., 2010; Zhang and Jiao, 2015; Estato et al., 2018). Therefore, we tested inoculums of 2, 5 and 10 cysts per pregnant female at E10 (Figures 1A, B). We observed that the 10- and 5-cysts inocula led to high lethality rates, reaching 100% death at E15 (or 5 days post infection) for 10 cysts (*N* = 2) and 25% for 5 cysts (*N* = 7), whereas inoculation with 2 cysts allowed 100% survival of pregnant female (Table 1, *N* = 13). However, the overall effects of *T. gondii* infection on offspring survival were noticeable. From a total of 33 pregnant mice with viable, accountable pregnancies followed until birth (being 13 controls inoculated with PBS, 13 with 2 and 7 with 5 *T. gondii* cysts), infected females had a higher litter loss rate. Such loss occurred due to miscarriage, cannibalism, stillbirths and resorptions (Supplementary Table 1), thus confirming gestational abnormalities (Simó et al., 2010). Given the reduced rates of viable post-natal pups, we decided to focus our analyses to E18 as this corresponds to the final steps of retinal ganglion and horizontal cell neurogenesis and the beginning of bipolar cells and rod photoreceptor differentiation in mice (de Campos et al., 2020).

Morphometric analyses were performed to determine the effects of congenital infection on the somatic growth of the offspring. *T. gondii*-infected pregnancies led to a reduction in embryo’s body size at E18 (3.5 ± 0.3 cm in controls and 3.2 ± 0.2 cm in infected, *p* < 0.0001) and E20 (3.85 ± 0.1 cm in controls and 3.45 ± 0.2 cm in infected, *p* = 0.0123). Body weight was decreased only at E20 (1.36 ± 0.07 g in controls and 1.15 ± 0.34 g in infected, *p* = 0.0152, Figures 1B–D) and no change was observed at E18. Placental development was also affected by maternal infection with *T. gondii*. At E18, placentas from infected embryos were larger (diameter of 0.89 ± 0.11 cm as compared to 0.81 ± 0.06 cm in uninfected controls, Figures 1E, G) but lighter (0.096 ± 0.02 g versus 0.12 ± 0.02 g in controls, Figures 1F, G). Maternal infection with *T. gondii* did not affect the number of fetuses/dam, since each pregnant female (either control or *T. gondii* infected) had an average of seven embryos at E18.

### Congenital *T. gondii* infection alters retinal neuroblast distribution

**FIGURE 2:**
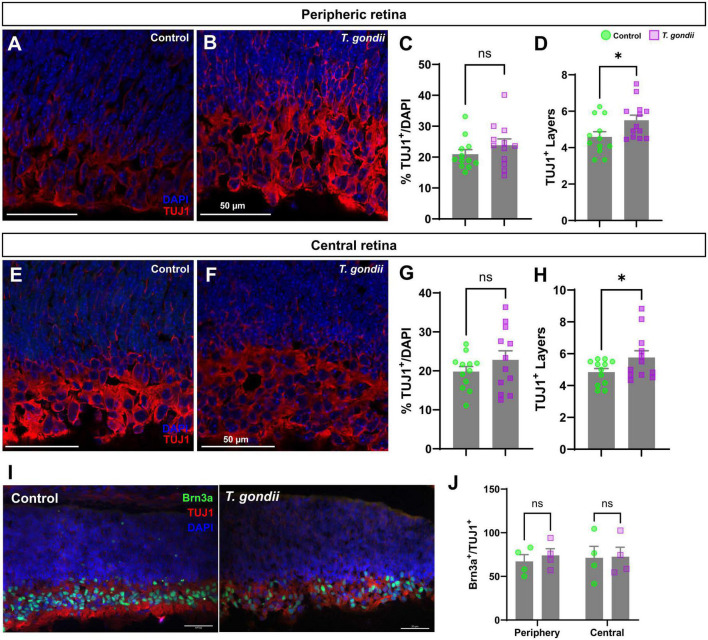
Congenitally infected embryos show increased TUJ1-stained area. Retinal sections of E18 embryos were immunostained for TUJ1 (**A,B** and **E,F**, in red). No changes in the number of TUJ1-positive cells in the infected offspring peripheric **(A–C)** and central **(E–G)** retinas was observed when compared to controls. However, a significant increase in the number of cellular layers occupied by TUJ1-positive cells was seen in the GCL region of the infected offspring in the peripheral **(D)** but not in central **(H)** regions of the retina. Specific RGC marker Brn3a was detected by immunohistochemistry [in green, in panel **(I)**]. No changes were found in the Brn3a + population within the TUJ1-stained area **(J)**. Results are shown from two independent litters, with three pairs of eyes each and nine images per slice. *p < 0.05, ns: not statistically significant, two-way ANOVA with Bonferroni post-test. Scale bar = 50 μm.

To assess whether CT could affect neuronal differentiation in the retina, we initially evaluated the thickness of retinal layers by confocal microscopy. No significant changes were found in GCL (Ganglion Cell Layer) and NBL (Neuroblastic Layer) in OT embryos at E18 as compared to controls, as determined by DAPI staining (Supplementary Figure 1). Next, we performed immunohistochemical analyses to detect the neuronal marker β-III-tubulin (TUJ1), one of the first markers of neuroblast-neuron differentiation (Zhang and Jiao, 2015). No statistically significant changes were observed regarding TUJ-1-positive cells, normalized by the number of nuclei, neither in the periphery (Figures 2A–C), nor in the central region of the retina despite the apparent trend (Figures 2E–G). However, when analyzing the overall distribution of TUJ1-staining, we observed an increase in the number of tiers labeling for TUJ1 by 1.22-fold in the periphery and by 1.21-fold in the central retina (*p* < 0.001 and *p* < 0.017, respectively, Figures 2D, H). Moreover, we quantified the number of Brn3a + /TUJ + cells (Figures 2I, J). Brn3a is a marker for retinal ganglion cells (Zhang and Jiao, 2015) and no changes to this neuronal population were observed in infected litters.

### Neural progenitor cell proliferation and mitosis are altered in retinas of congenitally infected mouse embryos

**FIGURE 3:**
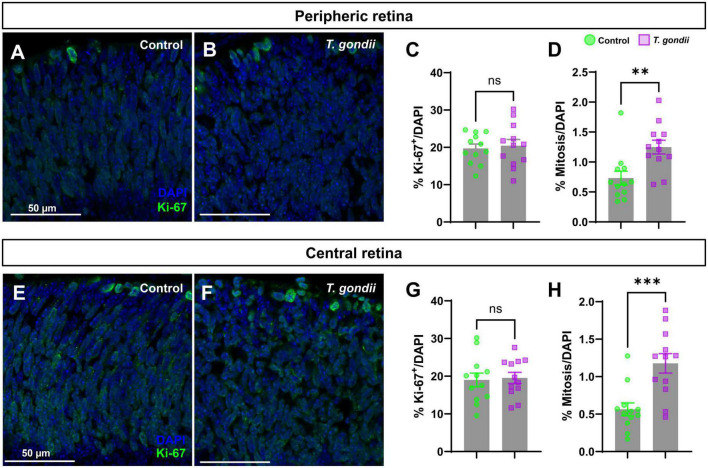
Congenital *T. gondii* infection causes an increase in retinal cells proliferation and mitosis. Retinal sections were stained for Ki-67, marker of cell proliferation and analyzed by confocal microscopy **(A,B,E,F)**. Ki-67+ cells at the apical side of the neuroblastic layer (in green) were considered as cells in mitosis. No statistically significant differences in the percentage of Ki-67-positive cells were observed between control and infected embryos **(C,G)**. There was a significant increase in mitotic cells in the central and peripheral regions of the retina of infected offspring **(D,H)**. Results are shown from two independent litters, with three pairs of eyes each and nine images per slice. **p < 0.01; ***p < 0.0001, ns: not statistically significant, two-way ANOVA with Bonferroni post-test. Scale bar = 50 μm.

The alteration in TUJ1 immunolabeling in CT retinas at E18 indicated a disruption in cell differentiation. It is widely established that cell cycle exit is a key step for neurogenesis (Ohnuma and Harris, 2003), determining the size of progenitor pool, cellular fate, neuronal number and cell subtypes (Ohnuma and Harris, 2003). To assess whether congenital *T. gondii* infection would affect the proliferation rate of retinal neural progenitors, we performed immunohistochemistry for Ki-67, a known nuclear protein preferentially expressed during the late G1-M phases of the cell cycle (Scholzen and Gerdes, 2000). We observed that the overall number of Ki-67+ cells remained unaltered both in the peripheral and central retinas of CT animals (Figures 3A, B). However, a significant increase of Ki-67+ cells in the apical region of the NBL, where cells in the M phase are found (Baye and Link, 2008), was observed in CT retinas compared to control ones, and this effect was consistent both in the peripheral (1.59-fold) and central (1.91-fold) regions (Figure 3).

**FIGURE 4:**
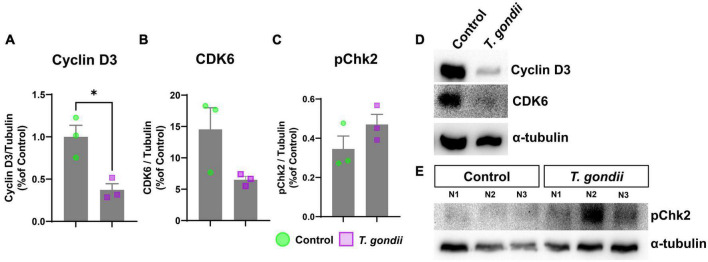
Abnormal cell cycle protein expression in congenitally-infected mouse retinas. Retinas from congenitally infected embryos or uninfected controls were collected at E18 and analyzed by western blotting to detect cell cycle proteins Cyclin D3 **(A,D)**, CDK6 **(B,D)**, and pChK2 **(C,E)**. **(D,E)**: Representative images of western blot for target proteins Cyclin D3, CDK6 and pChK2 and α-tubulin, used, as loading control. *: *p* < 0.05, unpaired Student’s *T*-test. Each point in graphs corresponds to one embryo.

Since CT had a selective effect on the mitotic cell population of retinal progenitor cells, we investigated whether such changes could be due to abnormal expression of cell cycle regulatory proteins. Retinas were collected and analyzed by western blotting to determine protein contents of Cyclin D3, and its target, CDK6, required for progression to G1 phase, and to stimulate the production of cyclin E, which ensures the progression from G1 to S phase (Hall, 1992). Both regulators, Cyclin D3 and CDK6 were significantly down-regulated in infected retinas, with 0.37- (*p* = 0.015) and 0.45- (*p* = 0.0826) fold change, respectively (Figures 4A, B, D). Finally, since *T. gondii* infection was shown to induce DNA damage (Velásquez et al., 2019), we also investigated the status of the checkpoint kinase 2 (ChK2), a protein activated by DNA damage and important to regulate the mitosis progression. Although ChK2 phosphorylation showed a 1.36-fold increase in infected retinas, it was not significantly different from controls (*p* = 0.2106) (Figures 4C, D).

## Discussion

Congenital *T. gondii* infection can lead to a broad spectrum of fetal malformations that can vary in severity according to the gestational period in which it occurs and can include miscarriages/reabsorptions, brain and eye abnormalities. Congenital anomalies significantly impact infant morbidity and mortality rates worldwide (Egbe et al., 2015). In Brazil, they represent the second cause of deaths in children under 1 year of age in all regions of the country, which corresponds to 22% of infant deaths (Guimarães, 2019).

The establishment of reliable experimental Toxoplasma congenital infection models that mirror the events observed in humans is challenging for several reasons. One of the difficulties is the balance between parasite virulence/mouse susceptibility, in a way that miscarriages of the offspring and/or death of the pregnant female occur within a controlled ratio (Wang et al., 2011). In this work, we proposed a model that allows studying congenitally *T. gondii*-infected offspring retinal development by using pigmented C57BL/6 mice. To assess the relationship between congenital infection by *T. gondii* and the damages to embryonic development, we used a model in which pregnant females were intragastrically infected with cysts of the ME49 strain of *T. gondii*, aiming to experimentally emulate the ingestion of contaminated bradyzoite cysts-bearing meat by pregnant female hosts. Our laboratory have described that intragastric inoculation of Swiss Webster mice with 25 or 50 Me49 cysts led to significant malformations to the skeletal muscle and brains and *T. gondii* cysts were found within fetal tissues (Gomes and Barbosa, 2017). We tested different cysts concentrations (2, 5 and 10) in pregnant females on the tenth day of gestation, in order to identify the most appropriate inoculum which would provide the best chance of post-infection survival, while still displaying significant fetal abnormalities. The inoculum of 2 ME49 strain bradyzoite cysts showed the best survival rate for pregnant mice, even though miscarriages, cannibalism, resorptions and stillbirths were still noticeable to a high degree. *T. gondii* infection during pregnancy can lead to serious consequences for the human embryo formation (Bollani et al., 1967; Hall, 1992; Hampton, 2015). Accordingly, our results confirmed the detrimental effect of congenital infection on development, as we observed a significant reduction in the weight and size of infected offspring compared to controls. Together, these data indicate that the animal model proposed herein is adequate to mimic human CT.

Several CT animal models have been proposed previously, using different species of rodents, mainly Swiss Webster albino mice strain, and different infection routes, such as intraocular, intrauterine, subcutaneous, neonatal and intragastric infection (Dukaczewska et al., 2015; Vargas-Villavicencio et al., 2016). Johnson (1994) demonstrated that intraperitoneal infection of pregnant female mice with 20 Me49 cysts led to 80% loss of infected offspring within 5 days after birth. Such mortality was correlated with cannibalism by the infected mothers, which was also observed in our model. In another study, inoculation of low amounts of oocysts by intragastric route led to high *T. gondii* levels in the brain (100%) and eyes (33%) of the surviving pups after birth. These data indicate that infection with lower inoculum by the intragastric route, mirrors more closely what is observed in humans, while maintaining viable offspring for further analysis (Müller et al., 2017). Similarly, other models using intravenous tachyzoites injection or intragastric cyst inoculation with type II strains (Me49 and Prugniaud, respectively) demonstrated macroscopic alterations, fetal death and cognitive impairment of the offspring (Wang et al., 2011; Vargas-Villavicencio et al., 2016). Importantly, albino mice display relevant alterations in retinal developmental, such as a temporal shift in neurogenesis, abnormalities in crossing of RGC axons at the optic chiasm and fovea formation. In adults, the RPE pigment (eumelanin and pheomelanin) improves optical quality by absorbing scattered light, recycles visual pigments, protects photoreceptor from blue light-induced damage and release important neurotrophic factors and protective retinal signaling pathways, among other important functions (Strauss, 2005). To model CT as closely as possible to the majority of the human population, which is not albino, it is important to use a pigmented animal strain.

Similar mechanisms of cellular alteration are shared by TORCH pathogens during congenital infections, including neural progenitor cell death, impaired neuronal migration, microcephaly, intracranial calcifications and other developmental brain and retina defects (Ostrander and Bale, 2019). We observed a significant increase in β-III-tubulin-stained area in retinas from infected dams, both in the peripheral and central regions of the retina. The production of cells that make up the neural retina occurs through the proliferation of the retinal population of neural progenitor cells and these proliferation and differentiation steps are partially overlapping. What differentiates the cells during these stages is, respectively, being in mitotic activity or having ceased it (Young, 1985a,b). Thus, the increase in differentiated neurons identified in *T. gondii*-infected offspring retinas indicates that infection could be causing cell cycle arrest. The elongation of RPCs proliferative stage is harmful to the development of the young retina, and may induce changes in the number and type of differentiated neuronal cells (Dyer and Cepko, 2001). Accordingly, Zika virus-infected neural progenitor cells were shown to display aberrant mitosis, with multipolar spindle, lagging chromosomes, micronuclei and death of the progeny after division (Souza et al., 2016). Furthermore, increased proliferation of late-born retinal progenitor cells after gestational lead exposure was shown to delay bipolar and rod photoreceptor cell differentiation (Chaney et al., 2016).

After observing an increase in neuroblast population in the GCL of infected offspring retinas, we analyzed the population of neural progenitors located in the NBL, where we verified a significant increase in the number of cells with mitotic profiles in the apical region of the retinas of infected offspring. Conversely, our group described that *in vitro* infection with tachyzoites of ME49 *T. gondii* strain leads to a decrease in the expression of Ki-67 in cortical neural progenitor cell cultures, which leads to decreased cellularity in a model of floating neurospheres (Pires et al., 2023). Changes in neural progenitor cell proliferation in the cerebral cortex and retina are seen in other types of congenital infections such as those by Herpesvirus and Cytomegalovirus (Tsutsui et al., 1993; Rotschafer et al., 2013; Kawasaki et al., 2017). Congenital rubella is another TORCH pathogen that is known to induce ophthalmic sequelae that may be correlated with sensory organs development-related gene expression regulation and with host cell cytoskeleton alterations, thus leading to mitotic pattern abnormalities (George et al., 2019). *T. gondii* infection changes cell cycle in different cell types, mainly in the G2/M transition phase (Brunet et al., 2008; Molestina et al., 2008). This dysregulation in the host cell’s cycle can be correlated with changes in key cell cycle proteins. Lavine and Arrizabalaga (2009) demonstrated that *T. gondii* infection in fibroblasts induces host cells to enter the mitotic S- phase, and that this transition may facilitate infection. Additionally, *T. gondii* infection of bovine endothelial cells leads to aberrant cytokinesis with marked effects on mitotic spindle formation (Velásquez et al., 2019). Soluble factors released from *T. gondii*-infected myoblasts exert a paracrine effect on rat cell cycle (Vieira et al., 2019), further corroborating the effects of infection on host cell proliferation rates. Therefore, the data shown herein demonstrates that gestational exposure to *T. gondii* disturbs the M phase of cell cycle in late retinal progenitor cells. It has been shown that the nuclear movement in interkinetic nuclear migration is tightly coupled to the cell cycle progression, and that the cell cycle progression likely regulates the activity of nuclear migration machineries (Kawauchi et al., 2013). Changes to the proliferating apical NBL cell counts (i.e., in M phase) could also indicate a disturbance in retinal progenitor cell cycle, a complex process, divided in phases, and modulated by several regulatory proteins (Kawauchi et al., 2013). D-type cyclins dictate the progression from G1 to S phase and, in mice, three Cyclin D genes are expressed: 1, 2, and 3 (Das et al., 2009). One of this study’s main goals was to describe a *T. gondii* gestational infection model and its effects on the developing retina. As initial mechanistic approach, we evaluated how maternal *T. gondii* exposure affects retinal progenitor cell biology, by focusing in cell cycle regulation. So, besides Ki-67 immunolabeling as a tool to assess proliferation, Cyclin D3 and cyclin dependent kinase 6 (CDK6), both involved in the regulation of G1 phase of cell cycle, were also investigated to show that not only the M but other phases of retinal progenitor cell cycle were affected. We demonstrated an expressive reduction of Cyclin D3 content, accompanied by a reduction of downstream Cyclin-dependent kinase 6 (CDK6) in the retinas of CT offspring. G1 phase appear to be important to determine the cell decision to divide or differentiate (Hardwick et al., 2015). For example, downregulation of cyclin-D-CDK4 lengthens G1 and increase neuronal differentiation (Lange et al., 2009). G1 phase has also been shown to regulate cell fate determination (Gao and Liu, 2019). We found a significant alteration in G1 phase regulators, CDK6 and Cyclin D3, which, together with the increase in the number of proliferating cells in the apical region, suggest an arrest in M phase in, at least, a population of retinal progenitor cells. CDK4 and Cyclin D1 overexpression in the developing mouse cortex shortens G1 phase, which delayed neurogenesis (Lange et al., 2009), whereas their downregulation induces a cell cycle lengthening (Kawauchi et al., 2013), further corroborating the relevance of our findings to establish the mechanisms by which *T. gondii* affects retinal neurogenesis. It was also shown that a G1-phase elongation increases the likelihood of daughter cells to exit the cell cycle (Tsuda and Lim, 2014).

Alterations in cell cycle proteins showed herein may be due to the expression of parasite proteins acting on cell protein expression. Some rhoptry proteins (ROPs) are known to affect host cell cycle progression (Brunet et al., 2008). Interestingly, different strains of *T. gondii* modulate different cell cycle protein expression (Molestina et al., 2008), which shows the parasite’s versatility to promote pathology. In addition to ROPs, dense granules proteins (GRAs) are also released and reach the host cell cytoplasm, regulating cell cycle and p53 tumor suppressor pathway-associated genes (Barnum and O’Connell, 2014), including GRA16, and GRA24 (Hakimi et al., 2017; Cygan et al., 2020). Cytomegalovirus alters host cell cycle, inducing quiescent cells to enter the cell cycle, but prevents them from entering the S phase, where host cell genome synthesis would compete with the virus for the available precursors for DNA replication, thus facilitating infection establishment (Kalejta and Shenk, 2002). We also analyzed the expression of the phosphorylated Checkpoint Kinase 2 protein (pChk2), which plays a critical role in the cellular response to DNA damage and is particularly important during the G2/M transition (Fragel-Madeira et al., 2011; Barnum and O’Connell, 2014). CT retinas showed a trend to increase, although not significantly, Chk2 phosphorylation levels. These observations open additional avenues for investigation of CT-induced changes in retinal progenitor cell biology.

## Conclusion

Cell cycle dysregulation is a mechanism that TORCH pathogens have in common. Whether forcing a stop at a specific phase, reactivating quiescent cells, inducing proliferation or differentiation, or even exiting the cell cycle. Such developmental changes can be harmful and irreparable, causing the morphological and/or physiological abnormalities found in human fetuses and neonates. In summary, we established an ideal model for studying changes in retinal development in congenitally-*T*. *gondii* infected pigmented mice. This model generated controlled lethality, with relevant changes in embryo’s body weight and length. Congenital infection induced a premature neuronal differentiation and increased pools of mitotic neural progenitor cells in the NBL, concomitantly with altered expression of cell cycle markers, thus indicating the arrest of these cells in the G2/M phase. Such alterations can influence cellular differentiation, leading to abnormal retinal formation.

## Limitations of the study

This work introduced important methodological aspects that should be taken into consideration in retinal toxoplasmosis studies. It does, however, still present limitations and points that could be improved in future studies. We recently optimized the husbandry protocol in our laboratory, to avoid manipulation of the pregnant females during the experiment, in order to reduce stressor stimuli. We found that cannibalism rates dropped considerably both in infected and control animals (da Rosa and Moreira dos Santos, personal observation), indicating that the protocol described herein was still not ideal. Female mice are now being kept separately after mating and are not marked in the tail throughout the experiment. We did not evaluate thoroughly the presence of *T. gondii* (either tachyzoites or bradyzoites) in retinal samples, or confirmed infection by any additional methods (i.e., parasite load in fetal brains or serological assessments), although immunohistochemistry for tachyzoite marker SAG1 yielded no parasite detection in our samples (not shown). The difficulty to detect *T. gondii* in the retina of congenitally infected animals was previously reported in the literature: *T. gondii* DNA was amplified in whole eye preparations from congenitally infected mice (Lahmar et al., 2010; Dukaczewska et al., 2015), but, to date, no attempt was made to amplify *T. gondii* DNA or RNA in isolated retinal samples; microscopical observation revealed parasitism in the retinal tissue in rabbits (Lee et al., 1983; Hay et al., 1984) with no direct demonstration of which cell types were harboring the parasites. As previously shown in a mouse model of congenital infection of albino mice by type II *T. gondii* strain, infectivity at P0 resulted in 5% of infected eyes, with minimal parasite load (Lahmar et al., 2010). These reports, combined with the data showed herein, suggest that further studies are needed to determine to what extent retinal damages are a result of direct parasitism of retinal cells (neuronal, glial or vascular cells), or of bystander effects of systemic inflammation. In fact, Hay et al. (1984) already pointed out for a possible role of systemic inflammation in the infected pregnant female cannot be ruled out, as cytokines and chemokines are known to cross the placental barrier and affect embryonic development (Lee et al., 1983; Hay et al., 1984).

## Data availability statement

The raw data supporting the conclusions of this article will be made available by the authors, without undue reservation.

## Ethics statement

The animal study was reviewed and approved by CEUA-IOC L-048/2015.

## Author contributions

VC, CM, BR, CS, and DF: formal analysis. DA and KC: conceptualization. VC, DA, CM, LF-M, and KC: data interpretation and methodology. All authors contributed to the article and approved the submitted version.

## Supplementary material

Supplementary Figure 1 Congenital *T. gondii* infection causes no change in retinal layer thickness at E18. In panels **(A–D)** are shown representative DAPI staining of control and infected retinas at different magnifications. We measured the ganglion cell layer (GCL), the neuroblastic layer (NBL) and the total retinal thicknesses in the peripheral **(E,F,G)** and central **(I,J,K)** regions. In panels **(H,L)** is the quantification of the cellularity analyses. Scale bars: 50 and 100 μm.ns: not statistically significant, unpaired Student’s *T*-test.
