# Supplementary material for: Maternal Toxoplasma gondii infection affects proliferation, differentiation and cell cycle regulation of retinal neural progenitor cells in mouse embryo
Source: Front Cell Neurosci. 2023 Jul 21;17:1211446. doi: 10.3389/fncel.2023.1211446 (PMC10400775; doi:10.3389/fncel.2023.1211446)
Supplement: Supplementary file 1 [file Table_1.docx]

**Table S1.** Pregnant female C57BL/6 mice followed in this study until the day of birth and their clinical outcomes.

| **Animal ID** | **Date of inoculation (dd/mm/yyyy)** | **Birth** | **Observations** |
| --- | --- | --- | --- |
| ***PBS group*** | | | |
| PBS 1 | 06/01/2019 | Yes | - |
| PBS 2 | 06/01/2019 | Yes | - |
| PBS 3 | 17/01/2019 | Yes | - |
| PBS 4 | 30/01/2019 | Yes | - |
| PBS 5 | 30/01/2019 | Yes | - |
| PBS 6 | 20/03/2019 | Yes | 9 pups |
| PBS 7 | 20/03/2019 | Yes | - |
| PBS 8 | 20/03/2019 | Yes | - |
| PBS 9 | 01/05/2019 | Yes | 5 pups |
| PBS 10 | 18/05/2019 | Yes | - |
| PBS 11 | 12/06/2019 | Yes |  |
| PBS 12 | 11/07/2019 | Yes | Cannibalism |
| PBS 13 | 18/08/2019 | Yes | - |
|  |  |  |  |
| ***T. gondii* – 5 cysts** | | | |
| CT 1 | 06/01/2019 | No | Mother died |
| CT 2 | 06/01/2019 | No | Mother died |
| CT 3 | 30/01/2019 |  |  |
| CT 4 | 17/01/2019 | No | Mother died |
| CT 5 | 17/01/2019 | No | Miscarriage |
| CT 6 | 24/02/2019 | No | Miscarriage |
| CT 7 | 24/02/2019 | Yes | One pup, stillbirth |
|  |  |  |  |
| ***T. gondii* – 2 cysts** | | | |
| CT 8 | 20/03/2019 | Yes | Cannibalism |
| CT 9 | 20/03/2019 | Yes | Cannibalism |
| CT 10 | 20/03/2019 | Yes | Cannibalism |
| CT 11 | 20/03/2019 | Yes | Cannibalism |
| CT 12 | 01/05/2019 | Yes | Cannibalism |
| CT 13 | 01/05/2019 | Yes | Cannibalism |
| CT 14 | 18/05/2019 | Yes | Cannibalism |
| CT 15 | 18/05/2019 | No | Mother died |
| CT 16 | 18/05/2019 | Yes | Cannibalism |
| CT 17 | 12/06/2019 | Yes | Cannibalism |
| CT 18 | 11/07/2019 | Yes | Cannibalism |
| CT 19 | 18/08/2019 | Yes | Cannibalism |
| CT 20 | 18/08/2019 | No | Reabsorption |
