# Supplementary material for: Maternal Toxoplasma gondii infection affects proliferation, differentiation and cell cycle regulation of retinal neural progenitor cells in mouse embryo
Source: Front Cell Neurosci. 2023 Jul 21;17:1211446. doi: 10.3389/fncel.2023.1211446 (PMC10400775; doi:10.3389/fncel.2023.1211446)
Supplement: Supplementary file 2 [file Table_2.docx]

**Table S2. Antibodies used in this study.**

| Antigen | Host | Target (relevant to this study) | Dilution factor | Source (Catalog number) |
| --- | --- | --- | --- | --- |
| Immunohistochemistry | | | | |
| Brn3a | Rabbit | Specific for Retinal Ganglion Cells | 1:1,000 | Synaptic Systems (411 003) |
| Ki67 | Rabbit | Nuclear marker of cells in cell cycle | 1:100 | Abcam (15580) |
| β-III-tubulin | Mouse | Specific isoform found in immature neurons | 1:200 | Cell Signaling (4466s) |
| Western Blot | | | | |
| CDK6 | Mouse | Detects endogenous levels of total CDK6 protein. | 1:1,000 | Cell Signaling (DCS83) |
| pChK2 ^Thr68^ | Rabbit | Detects endogenous levels of Chk2 only when phosphorylated at threonine 68. | 1:1,000 | Cell Signaling (2661) |
| Cyclin D3 | Mouse | Detects endogenous levels of total cyclin D3 protein. | 1:2,000 | Cell Signaling DCS22 |
| GAPDH | Mouse | Detects glyceraldehyde-3-phosphate dehydrogenase. | 1:4,000 | Thermo Scientific (258) |
| α-Tubulin | Mouse | Recognizes an epitope located at the C-terminal end of the α-tubulin isoform in a variety of organisms (load control). | 1:10,000 | Sigma (T5168) |
