# Supplementary figures and images for: Maternal Toxoplasma gondii infection affects proliferation, differentiation and cell cycle regulation of retinal neural progenitor cells in mouse embryo
Source: Front Cell Neurosci. 2023 Jul 21;17:1211446. doi: 10.3389/fncel.2023.1211446 (PMC10400775; doi:10.3389/fncel.2023.1211446)

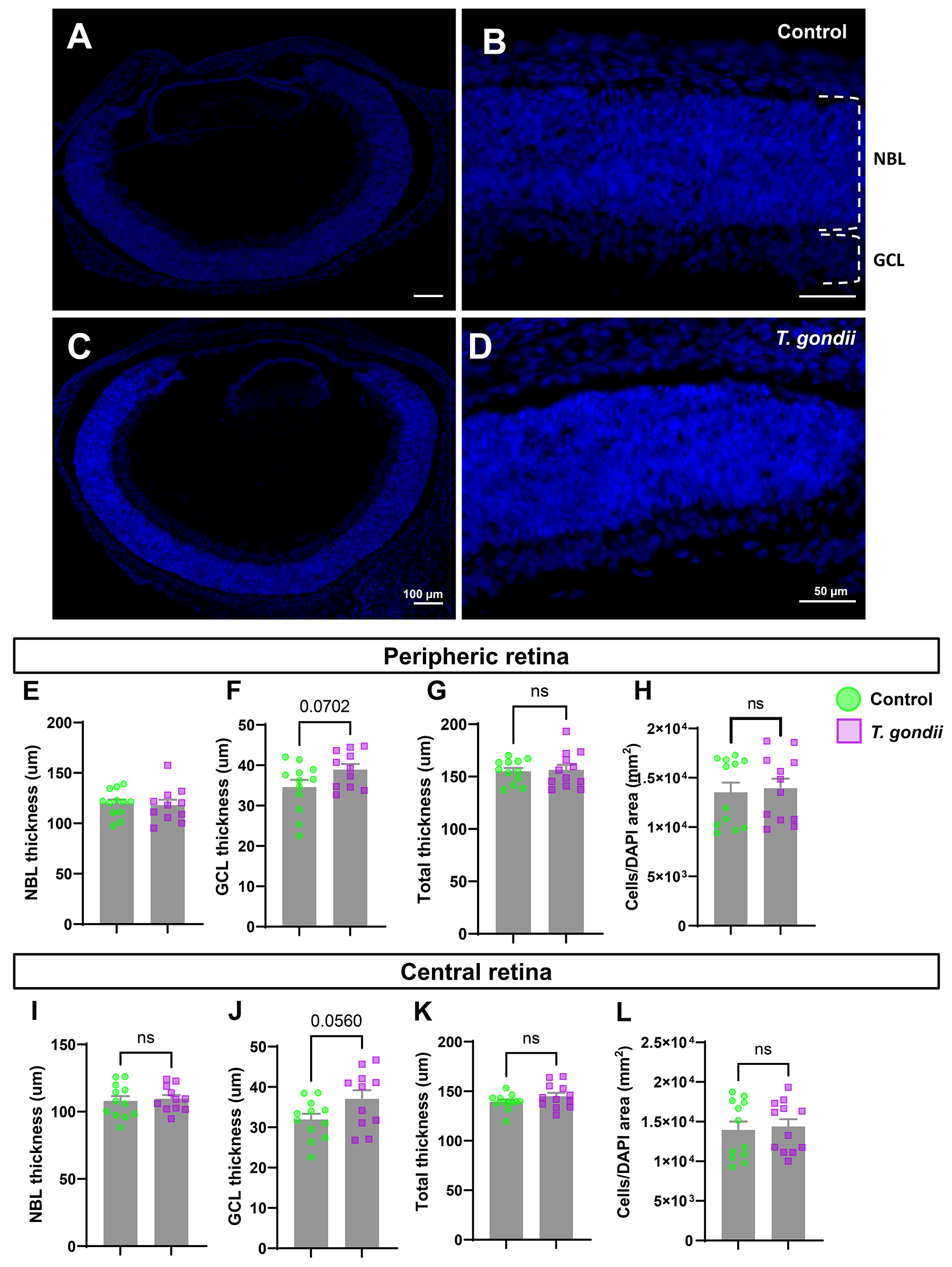

Supplement: Supplementary Figure 1 — Congenital T. gondii infection causes no change in retinal layer thickness at E18. In panels (A–D) are shown representative DAPI staining of control and infected retinas at different magnifications. We measured the ganglion cell layer (GCL), the neuroblastic layer (NBL) and the total retinal thicknesses in the peripheral (E,F,G) and central (I,J,K) regions. In panels (H,L) is the quantification of the cellularity analyses. Scale bars: 50 and 100 μm.ns: not statistically significant, unpaired Student’s T-test. [file Image_1.tif]
